# Intraoperative fluoroscopy improves surgical precision in conventional TKA

**DOI:** 10.1007/s00167-012-2350-6

**Published:** 2012-12-22

**Authors:** Hervé Hourlier, Peter Fennema

**Affiliations:** 1 Polyclinique de la Thiérache, Service d’Orthopédie, Rue du Dr Edmond Koral, 59212 Wignehies, France; 2 Biomet Orthopaedics Switzerland GmbH, Riedstrasse 6, 8953 Dietikon, Switzerland

**Keywords:** Total knee arthroplasty, Alignment, Randomized clinical trial, Fluoroscopy

## Abstract

**Purpose:**

The purpose of this study was to assess whether intraoperative fluoroscopy assists in the restoration of the coronal limb alignment target in conventional total knee arthroplasty (TKA).

**Methods:**

One hundred and six patients undergoing conventional cemented TKA were randomly assigned to be operated on with or without intraoperative fluoroscopy. The image intensifier, together with customized manual instrumentation, was used for separately measuring the frontal alignment of the femoral and tibial resection surfaces. The surgeon adjusted the resection surfaces when a mechanical axis deviation error angle of ≥0.5° was observed on the fluoroscopic image. Coronal alignment was measured on standing long-leg digital radiographs.

**Results:**

Patients operated with fluoroscopy assistance had (1) a lower risk of malalignment at the threshold of >3° (risk ratio, 0.7; 95 % CI, 0.13–1.2), (2) a mean fluoroscopic time of 3 s, and (3) a longer operative time (69 vs. 60 min, *p* < 0.001). The American Knee Society Score was not different between the two groups at 1-year follow-up.

**Conclusion:**

This new surgical intervention appears to offer an effective means for improving the precision of TKA alignment in the coronal plane.

**Level of evidence:**

Randomized clinical study, therapeutic study, Level I.

## Introduction

Coronal alignment has been identified as an important contributor to longevity in total knee arthroplasty (TKA) [3, 5, 13, 16, 18, 19, 21]. To decrease wear, it is recommended that the limb be aligned in such a way that the two compartments of the TKA are loaded evenly [10]. The postoperative mechanical axis of the lower limb should pass as a straight line through the centre of the hip (H), the centre of the knee (K), and the centre of the ankle (A). Aligning the prosthesis within 3° from this position (HKA = 180° ± 3°) is commonly accepted to be ideal. Conventional instrumentation systems have limited accuracy in determining the crucial landmarks (A and H) needed for alignment in TKA, with their use resulting in a malalignment of >3° varus or valgus in approximately a quarter of patients [8, 16]. The variability in bone geometry is the main cause leading to incorrect location of these landmarks [22]. In response, researchers have continued to look for alternative techniques that may remedy these hindrances. Computer-assisted surgery (CAS) and patient-specific guides (PSG) have been offered as potential remedies [6, 9, 17, 21]; however, these methods are associated with an increased burden in terms of costs, preparation and operative times, imaging requirements, and necessary equipment [20].

The use of intraoperative radiographs to locate the centre of the hip and the centre of the ankle during conventional TKA has been advocated as a plausible approach for improving component alignment [2]. Intraoperative fluoroscopy, together with the customized manual instrumentation, can potentially improve the accuracy of component alignment in TKA.

The primary objective of the current study was to investigate whether this method leads to more precise postoperative alignment in the coronal plane of the knee when compared with a standard technique. The secondary objective was to assess whether there were any differences in the duration of operation and early postoperative clinical outcome between the two groups. It was hypothesized that fluoro-assistance would result in enhanced total knee alignment in the coronal plane.

## Materials and methods

In 2008, 106 consecutive patients (64 women, 42 men; 107 knees) scheduled for primary conventional unilateral TKA were enrolled in this prospective, randomized, controlled study. Criteria for inclusion included pain and severe radiographic knee arthritis. There was no age limit for inclusion. Exclusion criteria included revision TKA and rheumatoid arthritis.

All patients received the same TKA design (cemented TC-Plus SB, Smith & Nephew, Baar, Switzerland). All surgeries were performed at the same centre by a single senior surgeon (HH).

Block randomization of the patients was performed after they had provided informed and signed consent. Allocation of patients to either group was made during the operation following the resection of the proximal tibia and the distal femur and after the two condylar holes were drilled. At the time of postoperative radiograph evaluation, neither patients nor the authors were aware of the group assignments.

Fifty-four (54) knees were operated using intraoperative fluoroscopy (group F) and 53 knees (group C) without it. Three patients were excluded from the primary analysis because they were unable to participate in long-standing radiographs within the period of three postoperative months. No remaining patients were lost in the early follow-up period (before 12 months). Therefore, 103 patients (104 knees) were available for the postoperative radiographic evaluation. The two groups were comparable for age and sex distribution. There were more valgus deformities recorded in group F (Table 1).

**Table 1:** Demographics and preoperative details

Parameter	Fluoroscopy group	Control group
Sample size (total knees)	52	52
Involved knee (right/left)	25/27	26/26
Age [years ± SD (range)]	74.8 ± 6.5 (58–87)	73.3 ± 6.7 (60–90)
Inflammatory knee condition (%)	5 (9.6 %)	5 (9.6 %)
Female gender (%)	25 (48 %)	27 (52 %)
Knees requiring patella resurfacing	9 (17 %)	8 (15 %)
Body mass index (kg/m^2^) (mean ± SD)	31.3 ± 6.5	30.3 ± 6.0
Preoperative mechanical axis (degrees) (mean ± SD) Varus/valgus/neutral	176 ± 9 38/11/2	173 ± 7 44/6/2

*SD* standard deviation

All patients were operated on a radiolucent table. A medial parapatellar approach with patellar eversion and tibial translation was used in all patients. Both cruciate ligaments were excised.

First, the tibia was prepared using an extramedullary alignment jig. The tibial cut was targeted to 0° in the frontal plane with respect to the mechanical axis and to a posterior inclination of 0° to 5° in the sagittal plane. For the latter, the native slope was used as a reference. The distal femur was cut by opening the femoral intramedullary canal. The entry point of the intrafemoral alignment jig was determined on preoperative long films. A distal femoral cutting jig was connected to the intramedullary rod by assembling a platform at 3°, 5°, 7°, or 9° valgus in relation to the anatomic–mechanical axis angle measured during preoperative planning. Intermediate (even) valgus angles were rounded up to the next (odd) value. The femoral cut was targeted to have the distal transverse plane cut at 0° with respect to the femoral mechanical axis in the frontal plane. The surgeon negotiated the femoral rotational alignment by compromising between the transepicondylar axis, which served as the primary reference line, and the anteroposterior axis of Whiteside [23] and by taking into account flexion balance.

Sizing, rotational alignment, and mediolateral position of the further femoral component were coordinately set using a unique instrument and reflected by drilling the two condylar holes used as base points. The ligaments were balanced using spacers. The patella was resurfaced at the discretion of the surgeon. CMW 3 Gentamicin bone cements (DePuy, Blackpool, United Kingdom), without the use of a pressurization technique, were used for all components.

In the fluoroscopic group (group F), intraoperative imaging was produced by a mobile C-arm fluoroscopy machine (Siemens, Erlangen, Germany) that was guided first over the hip region and later the ankle.

**Fig. 1:**
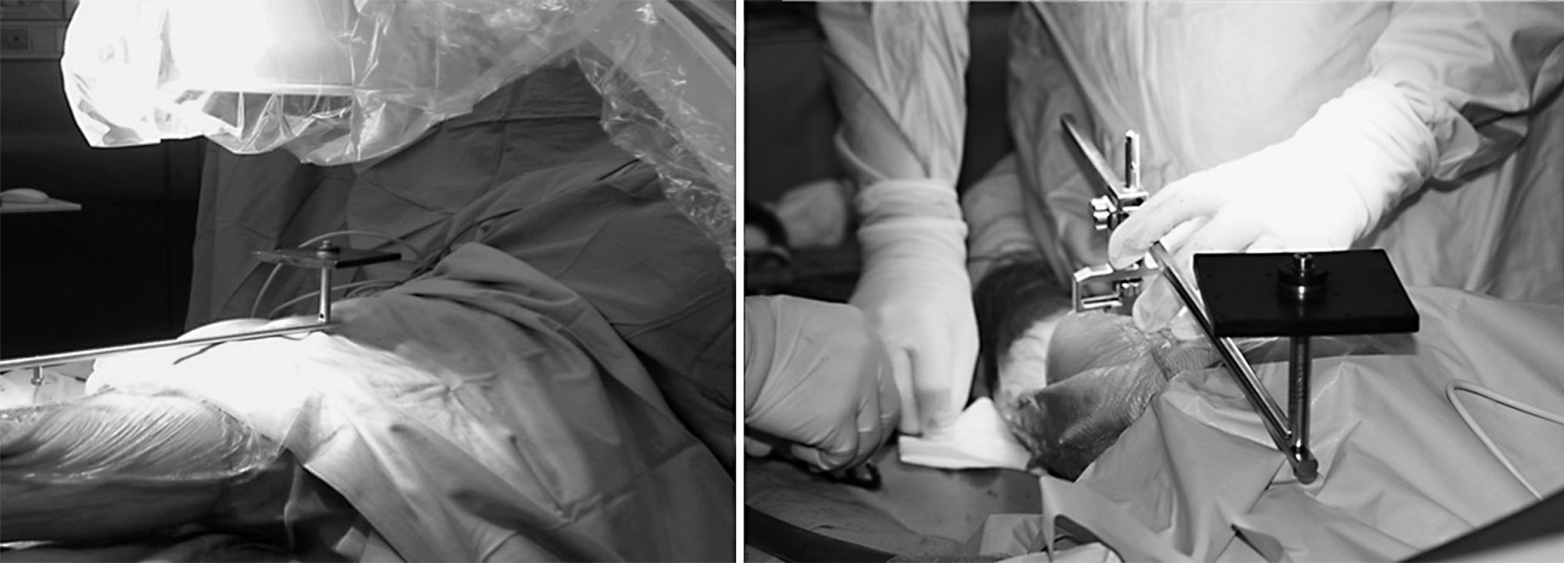
Aiming plate, attached by a screw to the extramedullary rod and connected to the base plate via a specially designed connector

**Fig. 2:**
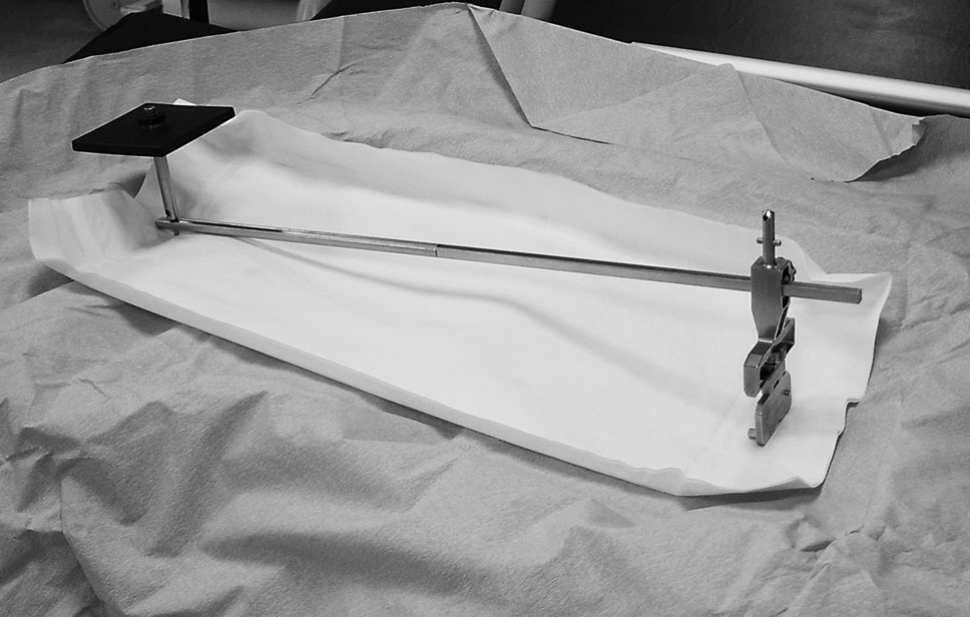
The extramedullary rod into the connector after placing the aiming plate in the hip joint region. The knee is extended

The radiographs were taken with the use of an aiming plate attached by screw under sterile conditions to an EM rod connected to a base plate via a specially designed connector (Fig. 1). A femoral base plate was specifically constructed for this study with a two-peg design intended to be inserted into the two condylar holes with the knee in flexion. Next, with the knee in extension, the surgeon screwed the EM rod into the connector after placing the aiming plate upon the hip region (Fig. 2). Thereafter, the receiver of the amplifier was guided by circulating personnel so that it was placed as parallel and close as possible to the aiming plate.

**Fig. 3:**
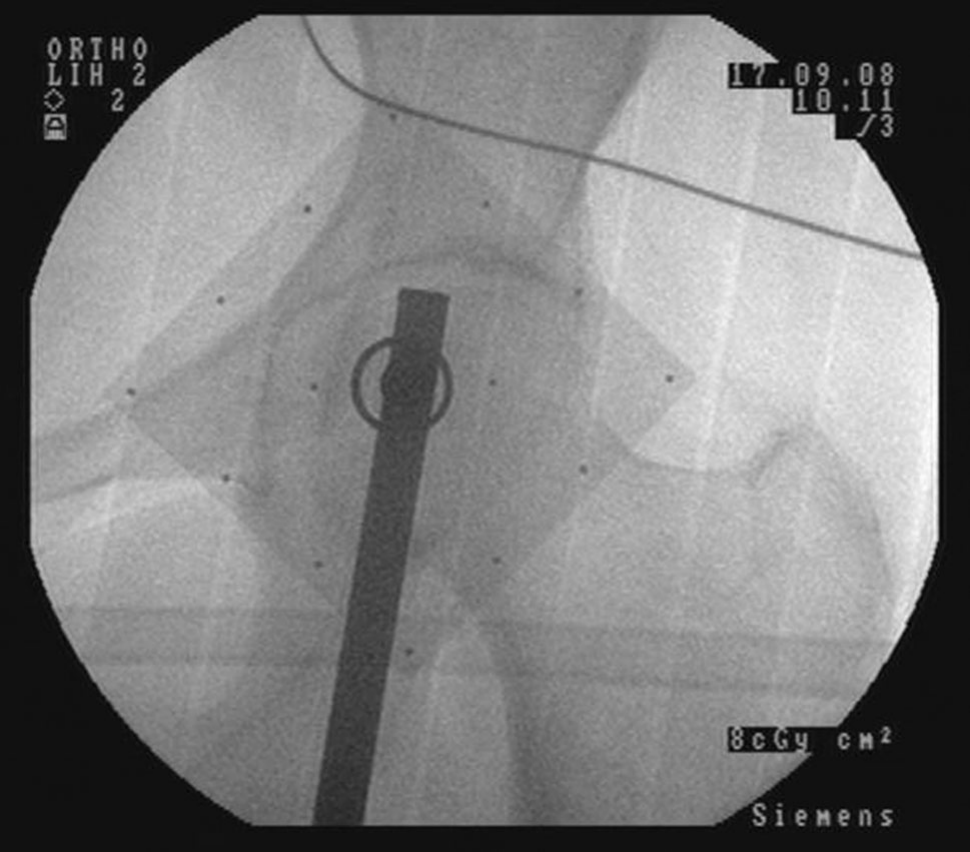
Fluoroscopic image showing correct alignment as shown by *extramedullary rod* in reference to the *centre* of the femoral head

The image delivered by fluoroscope (Fig. 3) was analysed by the surgeon in two steps. First, the surgeon observed the centring of the rod into the circle found on the aiming plate. A well-centred position for the rod on the horizontal diagonal of the circle indicates that the radiographic image was taken on a perpendicular direction upon the aiming plate and subsequently in the same perpendicular plane with the two condylar holes. If the rod was not observed at the centre of the circle, the C-arm was repositioned by circulating personnel and a new image was taken. Second, the surgeon analysed the fluoroscopic image for the position of the rod in reference to H. Any possible transverse distance between the rod and H was evaluated, and its magnitude was measured through the marker points found on the aiming plate. Normally, the distance between the two points corresponds to an angle of 2° when the receiver part of the C-arm is close to the aiming plate. When the deviation angle was >0.5° on the fluoroscopic image, the surgeon adjusted manually the bone resection using either a rasp or a 2° surface cut guide.

**Fig. 4:**
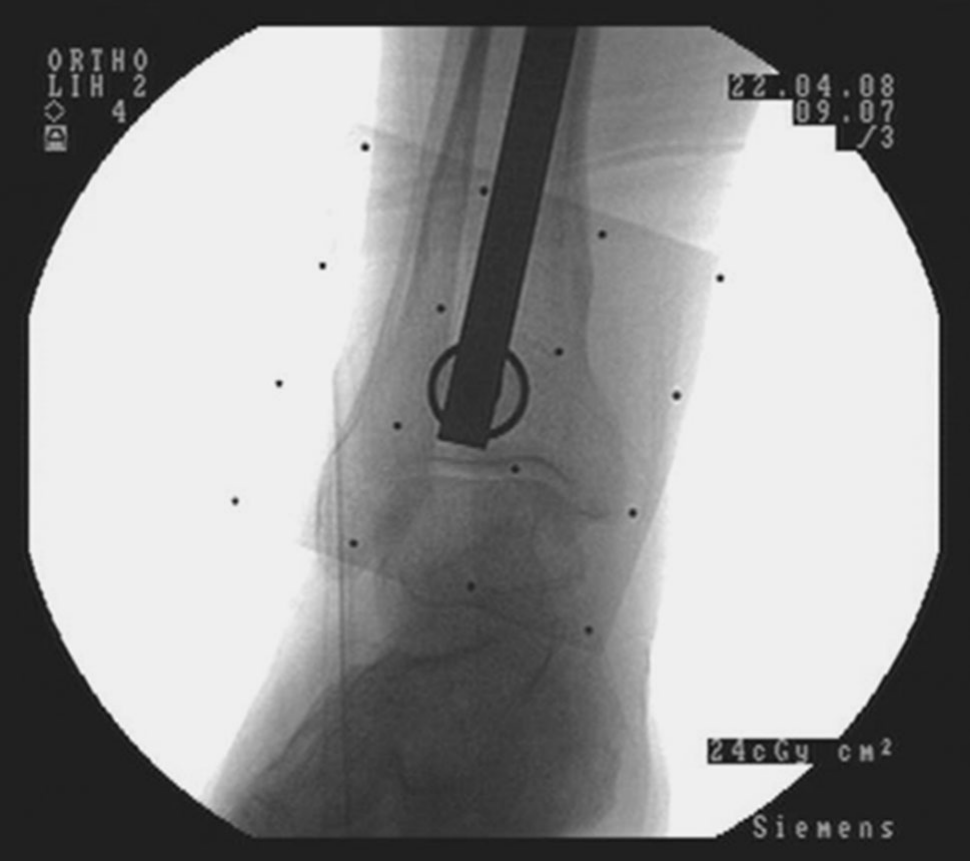
*Fluoroscopic image* showing correct alignment as shown by *extramedullary rod* in reference to the *centre* of the ankle joint

The frontal alignment of the tibia resection was measured with the trial prosthesis. The EM instrumentation previously used on the femur side was assembled to the original tibia base plate produced by the manufacturer. The alignment of the tibia base plate with the mobile insert was performed manually by the surgeon with the knee in extension. Next, the fluoroscopic image was realized on the ankle region (Fig. 4). The image delivered by fluoroscope was analysed by the surgeon in two steps in a similar manner as on the femur side. First, the surgeon observed the centring of the rod into the circle found on the aiming plate. If the rod was not observed at the centre of the circle, the C-arm was repositioned and a new image was taken. Second, the surgeon analysed the fluoroscopic image for the position of the rod in reference to the centre of the proximal part of the talus (A). Any possible transverse distance between the rod and A was evaluated, and its magnitude was measured through the marker points found on the aiming plate. Again, the surgeon performed adjustments on tibia bone resection when the deviation angle was >0.5°.

The following information was then recorded: (1) when and how often corrections were made on the bone resections in the fluoroscopic group, (2) the fluoroscopic time, (3) operative time, (4) the dose area product (DAP) as viewed on the screen of the intensifier, and (5) incision length.

Clinical and radiological evaluations were performed preoperatively and at 3 and 12 months postoperatively. Clinical evaluations included American Knee Society Scores (AKSS) [12]. Length of operation was defined as the time from incision to dressing and was recorded for both groups. Incision length was measured by the surgeon at the end of surgery with the knee in extension. Radiological examinations included digital standing full-leg frontal, skyline, and condylar radiographs.

The overall mechanical alignment of the knee before and after surgery was measured as the HKA (Hip–Knee–Ankle) angle on long-standing radiographs of the lower extremity according to a method described by Kim et al. [14] with the use of Photoshop (Adobe, San Jose, CA, USA). The method has a high intraobserver reliability and an interobserver agreement of 0.95 or more [14]. The radiology technicians were instructed on how to align the limb to obtain standardized full-leg radiographs with the patella pointing forward and the knee in maximum extension.

All the postoperative radiographs were performed 3 months postoperatively at the same centre by the same technician’s team. Measurements were recorded during full extension of the knee. Radiographs were retaken if they had inappropriate patient positioning (incomplete extension of the knee or any deviation from neutral rotation of the leg) or were of inadequate quality, as determined by the senior author (HH). Five radiographs in each group needed to be retaken. Radiographic data were examined by an independent blinded reviewer who had no knowledge of the patient’s group allocation.

### Statistical analysis

Univariate analysis was performed using unpaired parametric (*t* test) and nonparametric (Mann–Whitney, chi square, or Fisher exact) tests. Levene’s test was used to compare variances. Treatment comparisons for postoperative longitudinal continuous data were based on linear mixed models. The model included the preoperative level of the variable as an explanatory variable, the group-by-time interaction, and the main effects of group and time. A separate model was fitted for Knee Score, Function Score, and Flexion.

*p* values less than 0.05 were considered statistically significant. Data were analysed with Stata 11.2 (StataCorp LP, College Station, TX, USA).

The study was approved by the local ethics committee and partially supported by national public credit funds.

## Results

The mean postoperative HKA angle was 180° (range, 176–185°; SD, 2°) in group F and 179.9° (range, 175–186°; SD, 3°) in group C. The SD in group F was statistically significantly lower (*p* = 0.025, Levene’s test). The number of outliers >3° outside of the HKA range was 4 (7.7 %) in group F and 10 (19.2 %) in group C (*p* = 0.15, Fisher exact test). The frontal femoral component alignment was 90° ± 2° in group F and 90° ± 3° in group C (*p* = nonsignificant (n.s.)). The number of outliers >3° varus or valgus in the frontal component alignment was 5 (9.6 %) in group F and 10 (19.2 %) in group C (*p* = n.s.). The frontal tibial component alignment was 90° ± 3° in group F and 89° ± 3° in group C (*p* = n.s.). The number of outliers >3° varus or valgus deviation in the frontal tibial alignment was 6 (11.5 %) in group F and 11 (21.1 %) in group C (*p* = n.s.). The posterior inclination of the cemented tibia base plate component was 3° ± 3° in group F and 4° ± 3° in group F. In total, 29 of 52 knees in the fluoroscopic group had surgical adjustments of the femoral surface (range 1° to 2°), and 14 of 52 knees had adjustments of tibia alignment (range 1° to 2°). The fluoroscopic time varied from 2 to 6 s and DAP from 3 to 27 u Gy/cm^2^.

The mean duration of the operation was lower (*p* < 0.001) in the control group than in the fluoroscopic group (Table 2).

**Table 2:** Surgical variables

Parameter	Fluoroscopy group	Control group	*p* value
Operating time (min)	69.3 (12.6) [52–107]	59.2 (7.9) [41–77]	<0.001
Ischaemia time (min)	57.2 (12.6) [22–87]	46.8 (8.4) [30–70]	<0.001
Length of incision (cm)	17.8 (1.9) [12–22]	17.7 (2.0) [13–22]	n.s.

Values are expressed as mean, SD, and range

*n.s.* Non significant

No patients had intraoperative complications, and no procedures were aborted. Only one patient aged 89 years (group C) received a blood transfusion. At the time of 1-year follow-up, no patients had undergone revision of the total knee components or manipulation to treat stiffness. Flexion was slightly better in group F at 3 months (*p* = 0.001), but did not differ at the 1-year follow-up (*p* = n.s.) (Table 3). No significant differences were seen in terms of Knee Score or Function Score (Table 4).

**Table 3:** Flexion of the operated knees expressed in degrees preoperatively, at 3 months and at 1-year follow-up

Time period	Fluoroscopy group	Control group	*p* value
Preoperative	98 (94–102)	103 (97–109)	–
3 months	111 (107–114)	102 (98–105)	0.001
1 year	112 (108–116)	108 (194–113)	n.s.

Values are expressed as mean (95 % CI)

**Table 4:** Knee Society Knee Score and Function scores preoperatively, at 3 months and at 1-year follow-up

Time period	Fluoroscopy group	Control group	*p* value
KSKS			
Preoperative	22.5 (18.2–26.7)	25.9 (21.3–30.6)	–
3 months	87.1 (83.2–91.0)	87.0 (82.5–91.4)	n.s.
1 year	93.3 (89.0–97.7)	91.7 (86.7–96.7)	n.s.
KSFS			
Preoperative	41.7 (35.1–48.2)	45.0 (39.1–50.9)	–
3 months	57.0 (47.6–66.4)	57.9 (48.1–67.8)	n.s.
1 year	60.9 (51.4–70.5)	60.2 (50.2–70.3)	n.s.

Values are expressed as mean (95 % CI)

## Discussion

The most important finding of this study was that the use of intraoperative fluoroscopy leads to a significant reduction in variance in the postoperative HKA angle, when compared with conventional instrumentation. However, when measuring the number of outliers, the difference was not statistically significant, although the point estimate was still favourable for intraoperative fluoroscopy.

The best way to establish frontal alignment in TKA is still debated. Conventional jig-based techniques remain widely used in the orthopaedic community, yet have limited accuracy in locating the crucial landmarks (A and H) needed for exact alignment in TKA [16]. Because of variability in bone geometry, it is difficult to continually place knee prostheses within the target range of HKA 180° ± 3° in a single attempt without assistance. The use of intraoperative radiographs has been advocated as a possible means for accurately restoring limb alignment during conventional TKA [2].

In this pilot study, we investigated whether a new surgical intervention using intraoperative fluoroscopy, together with customized manual instrumentation, can help to achieve the coronal alignment target in TKA. The primary focus was to compare the mechanical leg axis after conventional TKA guided with and without intraoperative fluoroscopy. Our technique was initially developed to control the alignment of CAS with the mechanical axis in a single plane and produce an accuracy of <0.5°. To adapt this technique for conventional TKA, we created a femoral base plate with two pegs to use the two condylar holes as reference-based points. On the tibia side, we used the tibial base plate provided by the manufacturer for checking the frontal alignment of the tibial resection with the trial prosthesis in place. Because an inaccurate alignment between the tibial base plate and the insert with the knee in extension can affect the postoperative mechanical axis [1], we also developed an adaptor piece that connected the EM rod to the mobile trial insert, but this piece was not utilized for the present study in order not to alter the protocol study.

The current study was unable to prove the hypothesis that using intraoperative fluoroscopy leads to more accurate overall coronal alignment. Nevertheless, patients operated with the fluoroscopy guidance had a lower risk of malalignment (risk ratio, 0.7; 95 % CI, 0.13–1.2), which was in line with a meta-analysis showing similar results for patients managed with CAS (risk ratio, 0.79; 95 % CI, 0.71–0.89) [4]. The lower risk ratio was not significant though, given that the upper confidence bound crossed the 1.0.

Approximately 80 % of HKA angles were considered ideal in patients undergoing TKA with standard referencing classical techniques. This is the highest among such rates noted using a conventional instrumentation technique and is nearly identical to another series of conventional TKA performed by an experienced surgeon [14].

The use of intraoperative radiology for TKA is believed to be time-consuming and expensive and to carry a burden of additional radiation exposure [2]. Our study indicates that this particular approach is safe and reliable, with no discernible complications. There was no increase in the complication rate in the short term despite the mean operative time being longer by 9 min. The new surgical intervention did not require the placement of percutaneous pins in either the femur or the tibia. The mean fluoroscopy time was only 3 s, which is well below the 21 s reported by Victor and Hoste as being reasonable for image-based computer-assisted navigation [22].

To minimize the radiation exposure for the patient and the operating room personnel, fluoroscopy was used only during the control phase when bone cuts were performed, was not employed to check the results of the corrections performed by the surgeon, and was halted prior to final cementing of the implants. Moreover, the manoeuvring of the amplifier was greatly facilitated by the presence of a radiolucent table and the ergonomics of the aiming plate.

Numerous studies have suggested that CAS and PSG hold the promise for providing the most accurate intraoperative guidance for TKA [6, 17]. However, these technologies are not available to most surgeons, and their long-term relative economic benefit is currently being debated in this era of healthcare reform and cost control [8, 15]. Although a mobile image intensifier is undoubtedly a high-cost technology, it is nonetheless one that is readily available to the majority of orthopaedic surgeons.

A major strength of the current study is that it employed randomization, in combination with the blinding of the assessor of the primary study outcome, to minimize bias. The two study groups can be considered reasonably homogeneous at baseline. However, our study also has a number of limitations. Firstly, the jigs that were used are currently not readily available, which limits the general applicability of the described technique. Secondly, the study design did not allow for the evaluation of intersurgeon variability. All surgeries were performed with a conventional approach by an experienced arthroplasty surgeon using the latest generation of conventional jig-based instrumentation, and results may vary in a different treatment scenario. Thirdly, the decision was made to include only cemented components, in order to recruit the largest number of patients and thereby limit confounding variables potentially obscuring the interpretability of these results. Lastly, no formal sample size calculation was done prior to the study. Point estimates of several radiographic outcomes (e.g. outlier analysis of the HKA angle) showed relevant differences in favour of the study group, but the differences were not statistically significant. Additional studies that include surgeons from multiple settings and a larger sample size are therefore needed to confirm these results. Penetration of the cement may modify the final position of components [7]. Nevertheless, this limitation was shared by both the study groups. The study was also limited to plain radiographs, which are not as accurate as computed tomography (CT) scans. The extra exposure to more ionizing radiation associated with CT scans was also a consideration contributing to their exclusion in this study. Plain radiographs may be inaccurate if the patient’s extremities are rotated while the image is being taken, thereby potentially affecting the accuracy of the measurements; however, radiographs in the present study were performed using standardized guidelines and a digital, computerized method of measurement that has been found to reliably determine mechanical axis with a level of precision permitting the detection of differences <1° [11]. We were also unable to assess the rotational alignment of the components with plain radiographs.

## Conclusion

**Fig. 5:**
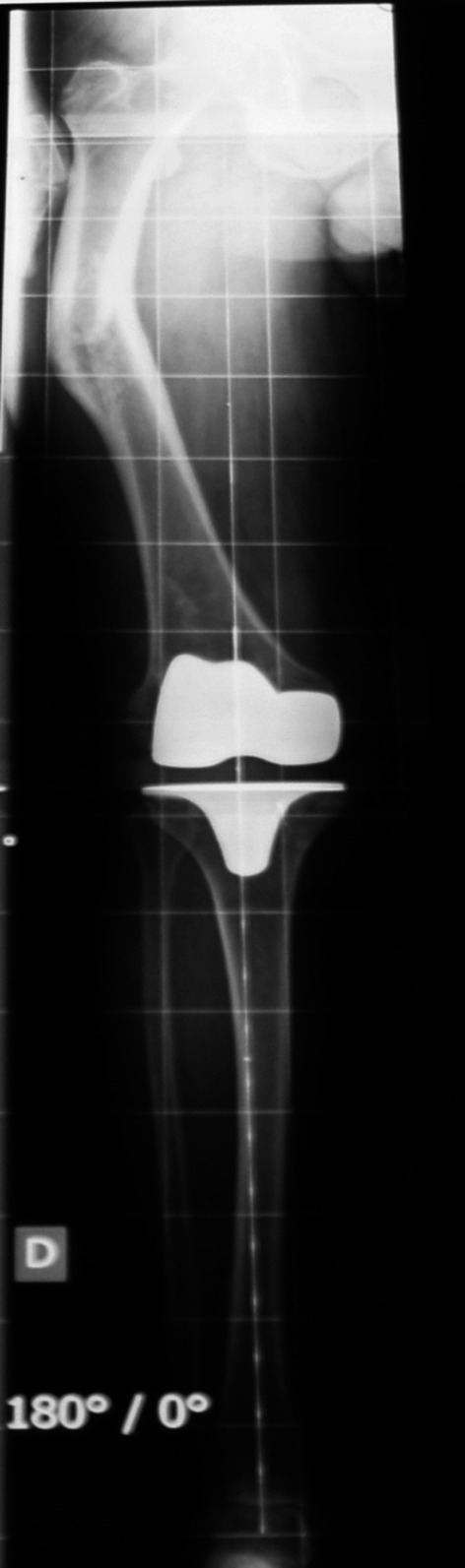
Correct postoperative alignment obtained by the intramedullary technique followed by adjustment by the 2° cutting guide

To conclude, our study showed that the use of this new surgical intervention led to improved precision of the prosthesis placement in the coronal plane, as determined by the HKA angle. In our opinion, the angle of the individual components displayed relevant improvement, although the difference was not statistically significant. We conclude this technology represents a safe and reliable surgical solution that can be routinely used in TKA surgery to improve mechanical alignment. Although not specifically assessed in our study, the technique may be particularly useful in patients with significant extra-articular deformities (Fig. 5) and/or intramedullary hardware.
